# The Optimal First-Line Therapy of *Helicobacter pylori* Infection in Year 2012

**DOI:** 10.1155/2012/168361

**Published:** 2012-06-27

**Authors:** Chao-Hung Kuo, Fu-Chen Kuo, Huang-Ming Hu, Chung-Jung Liu, Sophie S. W. Wang, Yen-Hsu Chen, Ming-Chia Hsieh, Ming-Feng Hou, Deng-Chyang Wu

**Affiliations:** ^1^Division of Gastroenterology, Department of Internal Medicine, Kaohsiung Medical University Hospital, Kaohsiung City 807, Taiwan; ^2^Department of Medicine, College of Medicine, Kaohsiung Medical University, Kaohsiung City 807, Taiwan; ^3^Cancer Center, Kaohsiung Medical University Hospital, Kaohsiung City 807, Taiwan; ^4^Department of Health Management, I-Shou University, E-Da Hospital, Kaohsiung County 824, Taiwan; ^5^Department of Public Health, College of Health Sciences, Kaohsiung Medical University, Kaohsiung City 807, Taiwan; ^6^Division of Infectious Diseases, Department of Internal Medicine, Kaohsiung Medical University Hospital, Kaohsiung City 807, Taiwan; ^7^Division of Endocrinology and Metabolism, Department of Internal Medicine, Changhua Christian Hospital, Changhua 500, Taiwan

## Abstract

This paper reviews the literature about first-line therapies for *H. pylori* infection in recent years. First-line therapies are facing a challenge because of increasing treatment failure due to elevated antibiotics resistance. Several new treatment strategies that recently emerged to overcome antibiotic resistance have been surveyed. Alternative first-line therapies include bismuth-containing quadruple therapy, sequential therapy, concomitant therapy, and hybrid therapy. Levofloxacin-based therapy shows impressive efficacy but might be employed as rescue treatment due to rapidly raising resistance. Rifabutin-based therapy is also regarded as a rescue therapy. Several factors including antibiotics resistance, patient compliance, and CYP 2C19 genotypes could influence the outcome. Clinicians should use antibiotics according to local reports. It is recommended that triple therapy should not be used in areas with high clarithromycin resistance or dual clarithromycin and metronidazole resistance.

## 1. Introduction

Eradicating *Helicobacter pylori* (*H. pylori*) is the most important aspect of managing *H. pylori*-related gastrointestinal diseases. In the past decade, the Maastricht III Consensus Report has recommended that proton pump inhibitor- (PPI-) clarithromycin-amoxicillin or metronidazole treatment is the first choice for *H. pylori* infection [1]. Although some studies have revealed that the eradication rates of standard triple therapies are around 80% (by per-protocol (PP) analysis) [2, 3], most studies have demonstrated the success rate of recommended triple therapies is falling [4–7]. According to recent studies, such eradication rates have plummeted to even 25%–60% [8–10]. The many causes of fall in efficacy are varied including antibiotic resistance, poor compliance, high gastric acidity, high bacterial load, and the cytochrome P450 2C19 (CYP2C19) polymorphism [10]. Compliance is an important factor where patients with good compliance (taking more than 60% of prescribed agents) have a higher treatment success compared to patients with poor compliance (96 versus 69%) [11]. The factors that negatively affect successful eradication are an increase in body mass index and smoking [12, 13]. Besides, other factors including the patient's history of antibiotic use, the cost, and availability of the drugs would also influence the choice of regimen.

In order to overcome the challenge of decreasing eradication rates, many novel first-line therapies have been developed. According to guidelines of the Maastricht III, the minimal acceptable eradication level recommended is an 80% eradication rate (by intention-to-treat (ITT) analysis) [1]. Graham and Fischbach recommended that clinicians should only use what works locally and ignore consensus statements and society guidelines if they are not consistent with local results [14]. According to the recommendation of the Asian Pacific *Helicobacter pylori* meeting 2012 in Singapore: (1) in areas with low clarithromycin resistance rates, standard triple therapy should be the primary choice, while bismuth-containing quadruple, sequential therapy, and concomitant therapy could be alternative first-line therapies and (2) in areas with high clarithromycin resistance, regimens including bismuth-containing quadruple, sequential therapy, and concomitance should be the better choice for first-line regimens. This paper will introduce recent novel and acceptable regimens as the first-line therapies of *H. pylori* and the factors influencing eradication.

## 2. Standard Triple Therapy

Triple therapies are still the most commonly used first-line therapy in the world despite decreasing efficacy [14]. Clarithromycin resistance plays the cardinal role in failure of eradication [14–16]. The standard triple therapy showed a better eradication rate in clarithromycin-sensitive strains than in clarithromycin-resistant strains (88% versus 18%) [16], so it is recommended that standard first-line therapies should be abandoned in areas with clarithromycin resistance of more than 15–20% [14], because the eradication rate often decreased to less than 85% (PP) and less than 80% (ITT) [8, 9, 15–17]. 

 However, prolonged duration of standard triple therapy might be a good method to overcome the challenge of resistance. A systemic review showed that the distribution of clarithromycin-resistant strains ranged from 49% (Spain) to 1% (Netherlands) worldwide [18]. One American study in 2011 surveyed the efficacy of 14-day triple therapy. The eradication rates of 14-day standard therapy, concomitant therapy, and sequential therapy were 82.2% (401 of 488), 73.6% (360 of 489), and 76.5% (372 of 486), respectively. It demonstrated that fourteen-day triple therapy is preferable to 5-day concomitant or 10-day sequential four-drug regimens [19].

## 3. Bismuth-Containing Quadruple Therapy

The Maastricht III Consensus Report [1] and the Second Asia-Pacific Consensus Guidelines for *H.  pylori* Infection [20] both recommended bismuth-containing quadruple therapy as an alternative first-line regimen for *H. pylori* infection. Three studies with this combination given for 10 days have demonstrated eradication (or successful treatment, but DC rates) rates more than 90% [21–23]. One study compared the efficacy of a 10-day bismuth-containing quadruple therapy and a 7-day triple therapy. Their data revealed that the bismuth-quadruple therapies had a higher eradication rate than the triple therapy (93% versus 70% by PP analysis) [23]. To improve compliance, one RCT presented that a capsule containing bismuth subcitrate potassium, metronidazole, and tetracycline given with omeprazole was comparable to clarithromycin-based triple therapy. The eradication rates were 80% in the quadruple therapy group versus 55% in the standard therapy group [23]. Besides, the bismuth-containing quadruple therapy provides superior eradication with similar safety and tolerability to standard therapy. So the quadruple therapy should be considered as first-line treatment in the areas of high clarithromycin resistance.

 However, there is no agreement with the duration of bismuth-containing quadruple therapy now. Ten or fourteen days are often used durations in these regimens [24]. Further survey is needed.

## 4. Sequential Therapy

A 10-day sequential therapy consists of a 5-day dual therapy with a PPI (standard dose, b.i.d.) and amoxicillin (1000 mg, b.i.d.) followed by a 5-day triple therapy with a PPI (standard dose, b.i.d.), clarithromycin (500 mg, b.i.d.) and metronidazole (500 mg, b.i.d.). This novel therapy shows an impressive eradication rate above 90% [25–28]. The rationale of sequential therapy includes (1) Amoxicillin would decrease the bacterial load and then the risk of selection of clarithromycin-resistant mutant and (2) Amoxicillin may disrupt the efflux pump preventing clarithromycin resistance. Gatta et al. reported a meta-analysis (8 Italian studies) [26] that compared sequential therapy with standard triple therapy for 7 or 10 days, and they found the relative risk of *H. pylori* eradication was 1.21 (95% CI 1.17–1.25). This meta-analysis showed a trend preferring sequential therapy to triple therapy. Sequential therapies also demonstrated better eradication rates than standard triple therapy for clarithromycin-resistant strains (89% versus 29% by PP analysis) [25].

However, there is significant heterogeneity observed between results from Asia and Italy. One study in Asia compared the sequential therapy with standard triple therapy and found that the two methods did not have significantly different eradication rates (86% versus 77% by PP analysis) [29]. This suggests that there is likely to be a variation in eradication rates achieved by sequential therapy in different areas. Another concern is the efficacy of sequential therapy for dual resistance (clarithromycin and metronidazole resistance). Unfortunately, there is still no large study to confirm this point. Besides, sequential therapy is more complex than triple or quadruple therapies and this raises the concern about patient compliance. However, one study stated that there was no significantly different compliance between sequential therapy and concomitant therapy [30].

## 5. Concomitant Therapy

 This regimen containing four-drug regimen: a PPI (standard dose, b.i.d.), clarithromycin (500 mg, b.i.d.), amoxicillin (1000 mg, b.i.d.), and metronidazole (500 mg, b.i.d.). All drugs are given during the course of concomitant therapy [30]. A meta-analysis was performed in 2009. It compares concomitant (293 subjects, duration 3 to 5 days) and triple therapy (283 subjects, duration 5 to 10 days) and four other studies evaluating concomitant therapy (478 subjects, duration 3 to 7 days). Pooled data showed that concomitant therapy had obviously better eradication rates than triple therapy: with pooled adds ratio (OR) of 2.86 (95% CI: 1.73–4.73) (by ITT analysis) and pooled OR of 3.52 (95% CI: 1.95–6.38) (by PP analysis) [31]. One recent study in 2012 also supports these results [32]. Concomitant therapy is less complex than sequential therapy. One randomized control trial compared the efficacy of sequential therapy and concomitant therapy and found that these two therapies showed similar eradication rates (93.1% versus 93.0% by PP analysis) and compliance [30].

## 6. Hybrid Therapy

 Hsu et al. [33] presented one novel therapy—The hybrid therapy. This therapy consists of two-step therapy: a dual therapy for 7 days (a PPI (standard dose, b.i.d.) and amoxicillin (1000 mg, b.i.d.)) followed by a quadruple therapy for 7 days (a PPI (standard dose, b.i.d.), amoxicillin (1000 mg, b.i.d.), clarithromycin (500 mg, b.i.d.), and metronidazole (500 mg, b.i.d.)). In this therapy, the role of fourteen-day amoxicillin is to reduce the bacterial load and try to overcome the challenge of *H. pylori* with dual resistance (metronidazole and clarithromycin). They demonstrated hybrid therapy with high eradication rates: 97% (by ITT analysis) and 99% (by PP analysis). This study also surveyed the efficacy in the treatment of *H. pylori* with dual resistance. It also showed encouraging results. Tests on the efficacy of this new regimen is needed with further studies.

## 7. Quinolone-Based Therapy

Levofloxacin could be used as an alternative agent for clarithromycin in either a standard triple, quadruple, or sequential regimens. The use of levofloxacin in first-line therapy has also been surveyed. The eradication rates of levofloxacin-based triple therapy ranged from 72% to 96% [34]. The variable rates may result from the difference in resistances. One study demonstrated efficacy of levofloxacin-based triple therapy had higher eradication rate than clarithromycin-based triple therapy (83% versus 66% by PP analysis) [35]. It also showed that levofloxacin-based quadruple therapy had similar eradication rates with clarithromycin-based quadruple therapy (85% versus 81% by PP analysis). Another study surveys the impact of levofloxacin on sequential therapy [36]. It demonstrated that levofloxacin-based sequential therapy had higher eradication rates than clarithromycin-based therapy (96% versus 81 % by PP analysis).

 The optimal dose of levofloxacin is another interesting point. The commonly used dosage of levofloxacin was 500 mg daily in many studies in Asia [37]. Studies demonstrated that increasing the dosage of levofloxacin cannot overcome levofloxacin resistance [38, 39]. Furthermore, previous studies suggest that once-daily dosing of a levofloxacin-based triple regimen may be as efficacious as twice daily [40].

One critical point should be remembered that quinolone resistance is raised rapidly in eradication of *H. pylori*. Primary resistance to levofloxacin ranges between 8% and 31% in different countries or regions [41–43]. Inappropriate use of quinolone might result in the development of more quinolone-resistant pathogens and it might cause much trouble in controlling respiratory (especially tuberculosis) and urogenital tract infections. So the quinolone-based triple therapy is not generally recommended as first-line therapy. The regimen could be considered in those areas with clarithromycin resistance greater than 15%–20% and quinolone resistance less than 10% [34].

## 8. Rifabutin-Based Therapy

Rifabutin is an antituberculous agent and it is also effective for eradicating *H. pylori* [44]. The optimal duration of rifabutin-containing therapies is unclear, but most studies have recommended 10–12 days. However, there are concerns about rifabutin-based therapies. One is the side effect of myelotoxicity (22% (19–25%)) and ocular adverse events have been reported with rifabutin-based therapy [45]. Another disadvantage is popular use of rifabutin might result in the development of resistance to Mycobacterium tuberculosis and Mycobacterium avium. So it is usually used in rescue therapies only.

## 9. The Factors Influencing Eradication of *H. pylori* Infection 

### 9.1. Resistance

Antibiotic resistance is the most serious problem in eradicating *H. pylori*. Resistance rates are remarkably variable in different geographic areas and therefore it is necessary to select the drugs according to local resistance patterns [46]. Clarithromycin resistance is the most important issue. The cause of high *H. pylori* clarithromycin resistance rates was mainly resulted from the long-term use of clarithromycin for respiratory tract infections [16]. A systemic review that included 11,697 cases was performed to survey the resistance rate of clarithromycin in the world in 2010. On a global scale, resistance was detected in 2014 cases (17.2%, 95% CI 16.5–17.9%). The resistance rates were obviously different among the following areas: Europe (11.1%), Asia (18.9%), and America (29.3%) [18]. A meta-analysis reported the impact of antibiotics resistance on treatment efficacy: clarithromycin resistance decreased the efficacy of PPI (standard dose, b.i.d.) + amoxicillin (1000 mg, b.i.d.) + clarithromycin (500 mg, b.i.d.) regimen by 66% (95% CI: 54–78%). The efficacy of patients receiving PPI (standard dose, b.i.d.) + metronidazole (250 mg, q.i.d.) + clarithromycin (500 mg, b.i.d.) regimen was decreased by 35% (95% CI: 24–44%) from clarithromycin resistance and decreased by 18% (95% CI: 13–23%) from metronidazole resistance [47]. Metronidazole resistance seems to have limited impact on efficacy of eradication.

 The resistance to metronidazole is between 30 and 40% [48, 49], although it has less clinical impact. Metronidazole resistance can be partially overcome by increasing the dosage or treatment duration.

 Resistance against amoxicillin is usually low around the world, so its resistance does not influence the use in treatment regimens.

Resistance to levofloxacin has increased rapidly in recent years and the worldwide resistance rate is around 16.2% (95% CI 14.4–18%). In Taiwan, about five-fold increase in levofloxacin resistance was observed in primary resistance (before the year 2004, 3.2%; after the year 2004, 16.3%) [49]. Average rate of primary levofloxacin resistance to *H. pylori* in Europe (2008-2009) is around 14.1% (4.0–28.0%) [47]. Resistance to fluoroquinolones would become a serious problem. The methods for preventing the selection of resistance include using a combination of antibiotics, increasing compliance, and increasing the length of treatment.

### 9.2. The Polymorphism of CYP2C19

The polymorphisms of CYP2C19 lead some patients to metabolize PPI more rapidly than others. Patients are divided into three phenotypes: extensive (EM), intermediate (IM), and poor (PM) metabolizers. Ethnic differences in the frequencies of CYP2C19 gene polymorphism are well known. Asian people have a higher proportion of poor metabolizers (20 versus 5%) compared to whites [50, 51]. The different phenotypes result in different degrees of PPI metabolism. The mechanisms whereby PPIs influence the efficacy of eradicating *H. pylori* include (1) PPIs make acid-labile antibiotics more stable by increasing gastric pH value, especially clarithromycin, thereby increasing concentration and *H. pylori* sensitivity to antibiotics (2) PPIs may alter transport of antibiotics from plasma to gastric juice, increase luminal concentrations and elevating the success rate of eradication [52]. CYP2C19 genotype-dependent *H. pylori* eradication rates were noted in many kinds of PPIs [51, 53, 54]. However, rabeprazole and esomeprazole were less influenced by polymorphism of CYP2C19 [51, 52]. 

The effect of increasing dose is unclear. One study in China demonstrated that increasing the dosage of omeprazole (20 to 40 mg) would improve the efficacy of eradication [55], but other studies did not find a similar dose-dependent effect by use of omeprazole, rabeprazole, and lansoprazole [56, 57]. 

### 9.3. The Impact of Probiotics in Eradicating *H. pylori*

It is difficult to develop new effective antibiotics to eradicate *H. pylori*, so it is necessary to find alternative methods to improve eradication rate and compliance in first-line therapy. So many studies have tried new treatment approaches by using probiotics. Several studies have previously reported that certain probiotics exhibit inhibitory activity against *H. pylori* in vitro and in vivo [58, 59]. Earlier studies demonstrated that standard triple therapy plus probiotics showed better eradication rate than standard triple therapy only [60–62]. So probiotics become a promising adjunct for *H. pylori* eradication therapy because they could increase compliance by increasing tolerability and preventing side effects [63–66]. The possible mechanisms of probiotics in eradicating *H. pylori* include production of inhibitory substance, host immune modulation or competition for adhesion [64, 67, 68]. But improvement of eradication rate is not always found in every regimen. One study revealed that levofloxacin-based sequential therapy and levofloxacin based triple therapy were significantly superior to standard triple therapy plus probiotic (PP/ITT analysis: 95.5/95.5%, 89.1/86.3%, and 77.1/72.4%, resp.) [69, 70].

 In previous studies, *Saccharomyces boulardii* and *Lactobacillus supp*. are the most common probiotics used in clinical trials. Several meta-analysis studies showed that standard triple therapy accompanied with the *Saccharomyces boulardii* or *Lactobacillus supp*. could increase eradication rates and decrease therapy-related side effects, especially diarrhea and taste disturbance [71–74]. 

 In summary, the exactly mechanism of probiotics is largely unknown and further research is greatly needed. The restoration of the normal flora in the intestine might be important in patients receiving triple therapy for *H. pylori* eradication.

### 9.4. Patients with Penicillin Allergy

Drug allergy to penicillin is also an important factor influencing regimen chosen. In *H. pylori* infected patients allergic to penicillin, the previously recommended first-line treatment with omeprazole-clarithromycin-metronidazole has low efficacy for curing the infection. So other regimens which include bismuth-containing, non-bismuth-conatining quadruple therapies or levofloxacin-based triple therapy should be taken into consideration. These regimens showed better and acceptable eradication outcomes [75]. So it is reasonable to choose quadruple therapy or levofloxacin-based triple therapy for patients allergic to penicillin. 

### 9.5. Smoking

 Smoking might cause failure of *H. pylori* eradication therapy. One meta-analysis of 5538 patients in 2006 revealed that the summary OR for eradication failure among smokers relative to nonsmokers was 1.95 (95% CI: 1.55–2.45; *P* < 0.01). It also showed a better eradication rate of 8.4% (95% CI: 3.3–13.5%; *P* < 0.01) in nonsmokers [13]. 

## 10. Conclusion

First-line therapies of *H. pylori* infection are facing a challenge because of increasing treatment failure. The paper reviews several new treatment strategies with the intention to overcome antibiotic resistance (Table 1). Alternative first-line therapies include bismuth-containing quadruple therapy, sequential therapy, concomitant quadruple therapy, and hybrid therapy. Levofloxacin-based therapies showed impressive efficacy, but they might be employed as rescue treatment except in areas with high clarithromycin resistance. Antimicrobial resistance is very important to clarithromycin-containing therapies because of their impact on clinical outcome and high prevalence. Antimicrobial resistance is not important for the other groups of antibiotics (amoxicillin, tetracycline) because of the low prevalence. However, it is not practical to perform culturing before first-line therapy. The impact of CYP2C19 polymorphism on eradication should be also taken into consideration. The following recommendations are important. (1) Clinicians should know the local resistance rates. (2) In areas with low clarithromycin resistance rates, standard triple therapy should be the primary choice, while bismuth-containing quadruple, sequential therapy and concomitant therapy could be alternative first-line therapies. (3) In areas with high clarithromycin resistance, regimens including bismuth-containing quadruple, sequential therapy, and concomitant should be the better choice for first-line regimens. In summary, *H. pylori* infection is a common and serious infection, and we should prescribe the first-line regimens more carefully and empirically. Clinicians should use antibiotics according to local reports. 

## Figures and Tables

**Table 1:** Recommended first-line therapies for *Helicobacter pylori* infection.

Treatment	Regimen	High clarithromycin resistance area	Low clarithromycin resistance area
Standard triple therapy	A PPI (standard dose, b.i.d.), clarithromycin (500 mg, b.i.d.), and amoxicillin (1 g, b.i.d.) for 7–14 days	x	V

Bismuth-containing quadruple therapy	A PPI (standard dose, b.i.d.), bismuth (standard dose, q.i.d.), tetracycline (500 mg, q.i.d.), and metronidazole (250 mg, q.i.d.) for 10–14 days	V	V

Sequential therapy	A 5-day dual therapy with a PPI (standard dose, b.i.d.) and amoxicillin (1 g, b.i.d.) followed by a 5-day triple therapy with a PPI (standard dose, b.i.d.), clarithromycin (500 mg, b.i.d.), and metronidazole (500 mg, b.i.d.)	V	V

Concomitant therapy	A PPI (standard dose, b.i.d.), clarithromycin (500 mg, b.i.d.), amoxicillin (1 g, b.i.d.), and metronidazole (500 mg, b.i.d.) for 7–10 days	V	V

Levofloxacin-based triple therapy	A PPI (standard dose, b.i.d.), levofloxacin (500 mg, q.d.), and amoxicillin (1 g, b.i.d.) for 10 days	V	—^∗^

Hybrid therapy	A 7-day dual therapy with a PPI (standard dose, b.i.d.) and amoxicillin (1 g, b.i.d.) followed by a 7-day quadruple therapy with a PPI (standard dose, b.i.d.), amoxicillin (1 g, b.i.d.), clarithromycin (500 mg, b.i.d.), and metronidazole (500 mg, b.i.d.)	V	V

*Levofloxacin-based triple therapy is useful, but it might not be recommended as first-line therapy under the consideration of rapidly increasing resistance.
